# The history of the two‐signal model of lymphocyte activation: A personal perspective

**DOI:** 10.1111/sji.12762

**Published:** 2019-03-18

**Authors:** Peter A. Bretscher

**Affiliations:** ^1^ Department of Biochemistry, Microbiology and Immunology University of Saskatchewan Saskatoon Saskatchewan Canada

**Keywords:** autoimmunity, cell activation, cells, costimulation, processes, T cells, tolerance/suppression/anergy

## Abstract

The first ideas leading to The Two‐Signal Model of lymphocyte activation were published 50 years ago, but the model was not realized in one sitting. I describe the three phases that led to its contemporary formulations. A motivation underlying all these models was to generate a minimal description of what is required for antigen to inactivate and activate mature lymphocytes that, at the same time, accounts for how peripheral tolerance is achieved. I suggest the two signal model has not only provided a substantiated framework for understanding how antigen interacts differently with B cells and CD8 T cells, to result in their inactivation and activation, but its postulates are pertinent to contemporary issues concerning the inactivation and activation of CD4 T cells.

## 1 INTRODUCTORY REMARKS

There are two related reasons for wishing for an accurate history of how scientific advances are made. The most obvious and prosaic is to give credit where credit is due. The second is more important and interesting. Knowledge of how important advances are made is germane to understanding how progress occurs. Such understanding can be empowering.

I clarify at the outset what different people mean by “two signal models” in the context of lymphocyte activation. There are two different and sometimes confusing uses. Dresser and Mitchison clearly stated in 1968 the idea that competent lymphocytes can interact with antigen in two ways, one way leading to their “paralysis”, or inactivation, and the other leading to their “induction” or activation.^1^ Lymphocytes can thus receive two distinct kinds of signal, each leading to different fates. I shall refer to this use as “a model for the two signals involved in the inactivation and activation of lymphocytes”. However, the term “two signal model”, as now commonly used, refers to a two‐signal model of lymphocyte activation. This model, first proposed by myself and Mel Cohn in 1970,^2^ postulates that the activation of a lymphocyte requires antigen to interact with its antigen‐specific receptor, generating signal 1, that, if generated alone for a sustained time, leads to the inactivation of the lymphocyte. The activation of the lymphocyte requires the generation of this signal 1, and a second signal, signal 2. A major impetus for the genesis of this two‐signal model of lymphocyte activation was to provide an understanding of how self‐nonself discrimination might be achieved,^2^ as outlined below. This was a time when it was not generally recognized, as also outlined below, that there are central and peripheral mechanisms of tolerance.^1^ The two‐signal model bears on the mechanism of peripheral tolerance. We have recently argued for its pertinence in addressing contemporary issues regarding the peripheral tolerance of CD4 T cells.^3^

## 2 HOW I CAME TO WRITE THIS ACCOUNT

This account was stimulated in part by Cohn's accounts of how the two‐signal model of lymphocyte activation came about. I think these accounts are inaccurate in some respects and so obscure the considerations underlying the initial proposal. They therefore undermine an understanding of the imaginative, analytical and observational considerations involved. A correct appreciation of these considerations might stimulate others to be brave, a quality that fosters creativity. My intellectual braveness, such as it is, has been fostered by appreciating that of others; an appreciation gained by closely following their intellectual journeys. This fostering led me to believe in the subtlety of argument. I felt I was part of a community that recognized sophisticated thought. I hope a correct account of the origin of the two‐signal model can help others to be brave and thus contribute to our culture. The most effective type of braveness is probably unselfconscious; it arises from intense engagement with a problem and a faith, nurtured by experience, that subtle considerations can yield enormous dividends.

Cohn wrote an invited article of reminiscences for the 1994 Annual Review of Immunology, entitled the Wisdom of Hindsight.^4^ His description of the origin of the two signal model of lymphocyte activation was considerably different from my recollections.

My primary aim, for most of my career, has been to do the best science I can, anticipating this would speak for itself. I found support for this course with my happiness at how Cohn and I had expressed our vision in the 1970 Science article,^2^ entitled A Theory of Self‐Nonself Discrimination, our most cited publication, outlining the grounds for the two‐signal model. When I have read it, decades later, I have had few misgivings. However, I recently decided to make an effort to get some immunological ideas, including those underlying our 1970 model, more broadly considered by the immunological community.

I started a year of administrative leave in June 2013. I reconsidered my priorities, particularly in view of my age of 70, and my frustration, over decades, that ideas, that I considered may be valuable to the field, were barely considered by immunologists. Some were pertinent to the two‐signal model, others to immune class regulation. I wrote two “Discussion Forums”,^5^, ^6^ describing my current thinking, for the Scandinavian Journal of Immunology.

The first, on the inactivation/activation of CD4 T cells, drew a long response from Cohn that was published.^7^ I therefore became more familiar 4 years ago with both some of Cohn's post‐1970 scientific proposals and also with his description of how the 1970 Two Signal Model of lymphocyte activation came about.^4^, ^7^ I became motivated, in view of Cohn's statements on the origins of the two signal model, to give my different perspective, with supporting evidence.

Cohn, in his 1994 “The Wisdom of Hindsight” article,^4^ had a section entitled “The Origin of a ‘Two Signal Model’ of the Self‐Nonself Discrimination”. This section starts off with:

Peter Bretscher joined my laboratory in 1967; he was, by way of background, an X‐ray crystallographer who had a passionate curiosity about immunology as well as a clear, crisp way of analysing complex problems. This made the difficult question of the self‐nonself discrimination an ideal one to answer; we were aware that it had to be analysed correctly if a next step was to be made in immunology.

“We had two prior formulations, those of Lederberg^8^ and Forsdyke^9^… Forsdyke gave us the barest hints as to what a competing theory would entail… Forsdyke proposed a situation where an interaction leading to a doublet [antigen binding to two distinguishable antibody sites of identical specificity in close proximity] results in inactivation, whereas one leading to a singlet leads to activation, in any given cell. … A two signal mechanism distinguishing self from nonself by each antigen‐responsive cell was required, and Bretscher and I set out to develop just such a theory.” I take Cohn to mean here a model for the two signals involved in the inactivation and activation of lymphocytes, not a two‐signal model of lymphocyte activation.

I now describe my recollections concerning the origins of the two‐signal model of lymphocyte activation. I apologize for going into considerable detail; such an exposition seems necessary to both understand the basis, and assess the legitimacy, of what I say. In addition, this model was not developed at a single sitting. Indeed, the formulations given by Cohn and myself started going in different directions, perhaps not unnaturally, when I left Mel's laboratory in 1972, as I shall indicate.

### 2.1 The beginnings of my involvement

I became interested in immunology as a graduate student, while doing research in protein X‐ray crystallography, at the UK Cambridge Laboratory of Molecular Biology. I started graduate studies in 1964. I was much more interested, from the beginning, in what was going on in the Division of Molecular Genetics than in that of Structural Studies, to which I belonged. Brenner and Crick jointly headed the former division. About five graduate students and postdoctoral fellows, primarily from this division, met regularly on Saturday mornings for a few hours to chat, with Sydney Brenner and over Nescafe, about diverse scientific topics. I joined my brother Mark, on becoming a graduate student, in attending these get‐togethers. Mark soon left for postdoctoral studies. I continued attending these get‐togethers.

One Saturday morning, in 1966, Sydney talked about a paper by himself and Cesar Milstein, on a model for “The origin of antibody variation”, just accepted by Nature.^10^ I thought about this model over the next week, and came to believe it implausible. The next Saturday I explained my reasons to Sydney. Sydney was marvellous. He considered what I said and clearly thought it interesting. He took my suggestion, that there were invariant amino acid residues in the variable region of immunoglobulin chains, to Cesar, a possibility unanticipated on and in weak conflict with their model. Cesar confirmed the validity of my proposal. This confirmation made the Brenner/Milstein model less plausible. This incident spurned my interest in immunology.

In time, I made use of the invitation, placed on a noticeboard outside Francis Crick's office, to discuss science. I knocked on Crick's door on at least ten occasions. One of my earliest immunological ideas is now obvious. However, at the time, Francis appeared to think it new and was kindly enthusiastic. Some reasonable people were then unsure about the plausibility of the Clonal Selection Theory. My ideas buttressed the plausibility of some of the critical features of this theory.

I supposed that, when antigen interacted with antibody receptors on an antibody precursor cell, there must be “an interaction sensing site” that “recognizes” this interaction via changes in the structure/accessibility of the antibody receptor. I considered the situation that would occur as the genes coding for immunoglobulin chains are generated, in evolutionary time or somatically. I considered that this “interaction sensing unit” must be complementary to an invariant part of the antibody molecule; if it was complementary to a variable part, there would have to be two “complementary” mutations, one in the antibody genes, the other in the gene encoding the interacting sensing unit, an unlikely event. Assume, as seemed plausible, that the “interaction sensing unit” was complementary to an invariant part of the antibody. In this case, if the cell had two chemically different antibody receptors, the cell could not distinguish which of the antibodies interacted with antigen, and so could not, for example, *uniquely *only activate the transcription of genes coding for the antibody receptor that had interacted with antigen. I was thus led to the proposition one cell “must” be committed to the synthesis of antibody of just one specificity. These considerations were obviously another way of making plausible some essential aspects of the Clonal Selection Theory. They formed part of the two papers Cohn and I subsequently published in Nature in 1968^11^ and in Science^2^ in 1970.

Crick encouraged me, once he understood what I was driving at. Francis could initially not understand what I was saying, given my nervousness. He recommended I put my ideas on one side of a page. I gave him my effort. The argument was dense, and as rigorous as I could make it. He in time understood my considerations. I remember the details of this interaction, as it was so important to me. Francis chastised me, in his jovial way, a style he employed to lessen the sharpness of his advice. I did not need to take his recommendation of one page so literally, and so make the argument so dense. I did not have to prove my proposal, just make it plausible. My interactions with Francis determined to a considerable extent my scientific fate.

On getting interested in immunology, I asked Cesar Milstein for advice on what to read. He suggested Burnet and lent me his copy of The Clonal Selection Theory of Acquired Immunity.^12^ I read some of it. However, I was quite ignorant and naïve. I found it in parts implausible, and so did not read it in toto. I clearly remember, though, one remark. Burnet pointed out that an antigen, in order to be immunogenic, that is, able to induce the formation of antibody, had to be a macromolecule. This caught my attention. Small molecules could interact with antibody. The inference was, perhaps, that the simple interaction of antigen with antibody receptors is insufficient to activate an antibody precursor cell. I surely gleaned from Burnet the importance of the attribute of self‐nonself discrimination. I must have also been introduced by Burnet to the idea that the immune system relies on the early presence of self‐antigens in development in ablating the ability to launch anti‐self immune responses. I also searched for and read other articles, particularly after I had an accident.

## 3 GETTING INTERESTED IN SELF‐TOLERANCE

This accident occurred in mid‐1966. I was using a chemical that reacted strongly with proteins and readily permeated skin. I wore gloves when using it. The tip of my right index finger started throbbing one night as I fell asleep, on a day I had used this chemical. The next morning the tip was white and quite without feeling. In the long run, the tip was surgically removed and skin from my arm was grafted onto the raw end. However, before this, during the days after the mishap, a sharp line developed between what I imagined was healthy me and the proteins of my finger tip, altered by reacting with this chemical and so becoming “foreign”. This personal experience stimulated my interest in how the immune system managed to respond to foreign but not to self‐antigens. My limited reading, or a conversation with Cesar Milstein, I now do not know which, led me to believe that antigen could interact with antibody precursor cells in two ways, one leading to the antibody precursor cell's inactivation, the other to its activation, that is, multiplication and the differentiation of its progeny into antibody‐secreting cells.

I had already decided to try to become an immunologist if I managed to get my PhD. In talking about this with Francis, he suggested I might do postdoctoral studies with Avrio n Mitchison, at that time at Mill Hill, London. I therefore visited Av, as he was known to all, probably sometime in 1966. Av explained his laboratory was very full but that, if I really wanted to come, he could probably squeeze me in.

## 4 THE ONE LYMPHOCYTE/MULTIPLE LYMPHOCYTE MODEL FOR THE ANTIGEN‐DEPENDENT INACTIVATION/ACTIVATION OF LYMPHOCYTES

I had some ideas, probably in 1967, and certainly after my meeting with Av, about how antigen interacts differently with antibody precursor cells to result in their activation and inactivation. I realized there were three appealing characteristics of the proposal that single precursor cells are inactivated by antigen, but that their activation requires the antigen‐mediated interaction between specific lymphocytes. Firstly, it was the only proposal I knew of that attempted to account for how antigen interacted differently with the same antibody precursor cell to result in either its inactivation or its activation. Secondly, it “explained” why antigens had to be macromolecular to be immunogenic. A small, univalent molecule, could not mediate the interaction between lymphocytes. Thirdly, it accounted for how self‐nonself discrimination could be achieved, as outlined elsewhere.^2^, ^3^, ^5^, ^13^, ^14^ Briefly, anti‐self lymphocytes were envisaged to be inactivated when they were first generated, one, or a few, at a time, due to the early presence of self‐antigens in ontogeny and their continuous presence thereafter. Lymphocytes only able to recognize foreign antigens would accumulate during ontogeny in the absence of the foreign antigen and, once the foreign antigen impacted upon the immune system, this antigen could mediate the lymphocyte cooperation needed to initiate an immune response. The ideas concerning the activation and inactivation of lymphocytes occurred to me before I had come across any observations on “carrier effects”.

I searched the literature, once I had formulated the one lymphocyte/multiple lymphocyte model, for observations bearing on it. I had no idea what journals to look at, so I randomly leafed through various periodicals on the library shelves. I found a paper in the Journal of Experimental Medicine that seemed to strongly support the proposal I envisaged. This was a paper from Benacerraf's group on “non‐responder” guinea pigs that did not produce antibody upon immunization with the antigen, poly‐L‐lysine (PLL). These animals produced anti‐PLL antibody when immunized with PLL coupled to an *immunogenic carrier*, namely bovine serum albumin (BSA).^15^ I thought that this inability probably reflected the existence of fewer PLL‐specific lymphocytes in non‐responder than in responder guinea pigs. I imagined there were more lymphocytes specific for the PLL‐BSA conjugate than for PLL in non‐responder guinea pigs. There was no interpretation in the paper along the line I envisaged. This was the first paper, among several, that seemed to support the ideas I had come up with, all associated with the so‐called carrier effects.

## 5 THE IMPACT OF TWO REVIEW ARTICLES

Two articles were very important to the further development of my ideas as a graduate student. Firstly, the article^1^ in the 1968 Advances in Immunology, by David Dresser and Av Mitchison, gave a very broad overview of observations and ideas pertinent to generating antibody responses and unresponsive states. Particularly important to me were the references to studies by Bill Weigle^16^, ^17^ from 1961 onwards, showing that immunization with an antigen, that crossreacts with the tolerogen, could break the unresponsive state, as described in more detail elsewhere.^2^, ^3^, ^5^, ^13^, ^14^ Weigle's observations fitted in with the proposal I was entertaining, and had been in the literature for several years. There were no suggestions I could find anywhere, along the lines I envisaged, for how these observations might be interpreted, for example in the Dresser and Mitchison article.^1^ This appreciation of the explanatory power of the ideas I entertained increased my perception of their plausibility and potential significance.

I also came across an article by Gell and Kelus, entitled “Anti‐Antibodies”, in Advances in Immunology.^18^ I was very interested in any conformational changes in the antibody molecule that occurred on interacting with antigen. Such changes had the potential for signalling to the precursor cell that its antibody receptor had interacted with antigen. I found the description, of antibodies that could recognize sites present on other antibody molecules that were complexed with antigen, but not present on unbound antibodies, very intriguing from this perspective.

I kept Francis informed on how my immunological ideas progressed. He changed his advice on where to do my postdoc. Francis was now annually visiting the Salk Institute, in southern California, as a Non‐Resident Fellow. Given his appreciation of my interest in theory, he suggested I might do a postdoc with Mel Cohn at the Salk Institute. Naturally, I accepted Francis' advice.

Mel Cohn also accepted me on Francis' recommendation. Cohn had a major, NIH‐sponsored, programme for producing myeloma tumours in BALB/c mice. He already had a substantial collection. I was encouraged to apply for a Damon Runyon Postdoctoral Fellowship while a graduate student in Cambridge. I had to outline a plausible research proposal. I proposed testing the hypothesis that myeloma proteins have an abnormal structure, reflecting a structure representative of antibody complexed with antigen. I hypothesized that if such abnormal “myeloma antibodies” were generated, in an antibody precursor cell, the cells might behave as continually activated, and so constitute a cancer of this cell. The prediction was that myeloma proteins, not complexed with antigen, would react with antibodies that normally only recognize antibody sites present on antibodies present in antigen/antibody complexes. Although this idea was not experimentally explored, as I came to feel it implausible once I had been in Mel's laboratory for a few months, it was part of my successful application for a postdoctoral fellowship.

Before leaving Cambridge for the Salk Institute, I submitted a letter^19^ to Nature on my thesis work. This letter was received by Nature on July 8, 1968, and subsequently published. I surmise I arrived in Cohn's laboratory in late July, 1968, at the very earliest.

## 6 ARRIVING IN MEL'S LABORATORY AND A HURRICANE OF EVENTS

My impression, on arriving at the Salk Institute, was that Cohn was primarily occupied with the problem of the origin of the diversity of antibody genes. He was less aware of some of the literature on the requirements to generate antibody responses and generate unresponsive states than I was. I in turn had not come across, as a graduate student, Lederberg's classic Science paper of 1959.^8^ I also became aware for the first time of the proceedings of the 1967 Cold Spring Harbour Meeting. The two major “new” discoveries for me, from reading the proceedings of this meeting, was that others, namely Mitchison,^20^ Rajewsky,^21^ and Jerne in his Summary of the meeting, had come to the view that the generation of an antibody response most probably required the antigen‐mediated interaction between lymphocytes. Naturally, I felt scooped. Secondly, Rajewsky's contribution described observations, novel to me, concerning his work with porcine lactic dehydrogenase (LDH) isozymes as antigens in rabbits. Rajewsky's observations were of particular interest to me. They were interpreted as showing that non‐immunogenic molecules were non‐tolerogenic, ie unable to induce unresponsiveness,^21^ a proposition at odds with the ideas I had developed in Cambridge. I thought this conclusion misleading, for reasons we outlined in our 1968 Nature^11^ and 1970 Science^2^ papers, and that I have also recently discussed.^5^

I was very motivated, on arriving in Cohn's laboratory, to review the literature; to see how my ideas stood up to current findings and other people's conjectures, and to explore how they might be further developed before “going public”. This was not to be.

Mel told me, shortly after my arrival, that he had been invited to a small meeting of about 40 individuals, on Immunological Tolerance, to be shortly held at Brook Lodge, Michigan. Moreover, Mel was asked to summarize the meeting. The invited participants included many leading investigators, including Av Mitchison, Klaus Rajewsky, Bill Weigle and Baruj Benacerraf. The meeting was held from September 18 to September 20, 1968, surely <2 months after I arrived in Mel's laboratory. It was natural for Cohn, in his summary of the meeting, to try to account for major findings in terms of the ideas we were developing.

Cohn's description of some reported observations was challenged by other attendees at the meeting, as evident from the original transcript, a copy of which Mel gave me. In particular, Cohn was unclear about the nature of Weigle's observations that showed that immunization with an antigen, that crossreacts with the tolerogen, could break the unresponsive state.^16^, ^17^ These findings critically supported our proposals, and were not explained by other, then contemporary, ideas. Our most contentious proposal, judging by the reactions of those at the meeting, was that non‐immunogenic molecules could cause unresponsiveness. Most thought there were carrier effects not only in generating an antibody response, but also in generating unresponsive states, or in causing the inactivation of the antibody precursor cell.^22^ Cohn referred to this analysis on our part, in his Wisdom in Hindsight article, in a way that seems historically incorrect and to reflect an unawareness of the paradoxes of the late 1960s. He said: “The second choice [of a requirement for recognition of a carrier for paralysis] was initially rejected on experimental grounds (ie recognition of the carrier is required for induction, not unresponsiveness).^4^” This statement misses the critical point then evident to me. Those who had come to grips with carrier effects, namely Mitchison and Rajewsky, believed that lymphocyte cooperation was required to induce antibody responses, but *argued that the evidence showed there were also carrier effects in generating unresponsiveness*. Cohn, in his recounting the origin of our ideas,^4^ misses the essential clarification our proposals brought about. It required careful, subtle and quantitative analysis to realize why the observations reported by, for example, Rajewsky, did not mean that there were carrier effects in the inactivation of antibody responses, as I have carefully recently recalled.^5^, ^13^

Both Cohn and I were uneasy about how Cohn had presented our ideas at Brook Lodge. Moreover, the proceedings of the meeting were to be published as a book.^22^ We decided in the circumstances to write an account of our ideas, as then formulated, as a paper, to be submitted to Nature.^11^

I received a personal letter from Av Mitchison, shortly after our Nature paper was published. He said “I think you deeply cloud the issue by using the carrier effect to account for the immunity/tolerance decision. I do not at all see why one cannot have all sorts of lovely carrier effects and also something else, for example, non‐specific stimulation from macrophages” (to induce an antibody precursor cell). Klaus Rajewsky had come to the Salk Institute straight from the Brook Lodge Meeting to talk science, a decision taken on the spur of the moment, as I understood at the time. I recall Klaus' uneasiness about the one lymphocyte/multiple lymphocyte model. As clear from our Nature and Science papers, we disagreed with a conclusion Klaus had come to in his studies in rabbits with porcine LDH. His observations were a particularly clear example of those employed by immunologists to support the proposition that *non‐immunogenic molecules were also non‐tolerogenic*. We argued that such observations were likely misinterpreted, as quantitative considerations^2^, ^3^, ^5^, ^11^, ^13^ could be employed to suggest the observations were not against the ideas I had formulated as a graduate student. Moreover, other observations supported these ideas.^2^, ^3^, ^5^, ^11^, ^13^ Furthermore, it seemed most plausible that most self‐antigens were *tolerogenic but not immunogenic*, in accordance with the one lymphocyte/multiple lymphocyte model.

This description gives my best recollection of the events leading up to the publication of our Nature paper, representing the first phase in the development of the Two Signal Model.

Our paper was submitted to Nature on October 17, 1968, less than a month after the Brook Lodge Meeting. It was published in the November 2, 1968, issue. I had been in Cohn's laboratory for <4 months, probably closer to two. Our Nature paper pretty well reflected the ideas I brought with me, except that there were references to the ideas of Mitchison, Rajewsky and Jerne on cell cooperation in the generation of antibody responses, and to Rajewsky's observations on tolerogenicity, referred to above. We (meaning I) thanked Crick for discussions related to this paper. Cohn was 46 and I was 25.

Cohn said, in his 1994 Annual Reviews article,^4^ that I came to his laboratory in 1967, but I came in late July, 1968, at the earliest. He talked about the influence of Donald Forsdyke's paper,^9^ published in the Lancet in early 1968. This is a rarely quoted paper that I first came across when reading Cohn's 1994 Annual Review article. Forsdyke's paper does not refer to any studies on carrier effects, central to our ideas. Cohn's account neglects to state that I came to his laboratory with the one lymphocyte/multiple lymphocyte model. Cohn's reference^4^ to Forsdyke's paper^9^ represents an enigma for me. I cannot imagine that, with all the intense discussions we had almost every day, that Mel would never have mentioned this paper if he had regarded it as important at the time.

I have never met Donald Forsdyke, but he emailed me in 2002 over some matter. When he emailed me, I felt I had to let him know of my ignorance of his 1968 Lancet paper when we formulated our ideas.^11^ Cohn had inferred^4^ we were inspired by his paper, an account taken up by some philosophers/historians of science.^23^ This account obscures the grounds for our original proposal. Moreover, if influenced by Forsdyke, we surely should and would have acknowledged it in our Nature paper. Thus not only is Cohn's account questionable, from my perspective, but his account makes it appear that Cohn and I were amiss in not giving credit where credit was due. I might add that very recently Forsdyke has given an account of the history of his model, entitled “Two signal half century: from negative selection of self‐reactivity to positive selection of near‐self reactivity”.^24^ Forsdyke's two signals represented one for lymphocyte inactivation, and one for lymphocyte activation. His model did not address the nature of carrier effects.

### 6.1 The three phases in the development of the Two Signal Model

I think it helpful if I explain at this point the three distinct phases in the history of the Two Signal Model of Lymphocyte Activation, as I perceive it. The first phase was the one outlined above, that culminated in our November 1968 Nature paper.^11^ The second phase reflected our joint discussions and deliberations over almost 2 years, when I was a Postdoctoral Fellow. This phase culminated in the 1970 Science paper^2^ outlining the Two‐Signal Model of Lymphocyte Activation. The third phase reflects different variations of two signal models that were put forward by Cohn, myself and others, over more than four decades, after I left Cohn's laboratory in 1973.

### 6.2 The second phase

The 1968 Nature paper was written fast and under considerable pressure. We were very soon uneasy with part of its content. I consider its strengths to be its articulation that antibody precursor cells would be inactivated upon interacting with antigen alone, and that their activation required a second site be recognized by antigen‐specific “carrier antibody”, derived from or present on another antigen‐specific cell, that is, a lymphocyte. However, its weakness was the envisaged mechanism by which the activation of the antibody precursor cell was “made to depend” on the recognition of a second site on the antigen by the carrier antibody. We suggested that the divalent carrier antibody held the antigen in a way that, when the antibody receptor of the antibody precursor cell bound to two bound antigen molecules, the receptor took on a “distorted” conformation, recognized by an “interaction recognition unit” of the cell. This proposal reflected ideas in my research proposal for a Postdoctoral Fellowship, written when I was in Cambridge. The model predicted that self‐antigens, with repeating epitopes that could distort the antibody receptors in the appropriate way on interacting with them, would induce autoantibodies, an awkward and implausible conclusion. The recognition of this weakness led to the second phase.

The “Miller papers”,^25^, ^26^ confirming that the generation of an antibody response requires cooperation between bone marrow (B) cells and thymocytes (T cells), and demonstrating that the B cells were the antibody precursor cell, came out in October, 1968, the month previous to that in which our Nature paper appeared. These papers were based upon a previous 1966 study by Claman et al,^27^ the first study to demonstrate that optimal antibody responses require cooperation of bone marrow cells and thymocytes. Miller's papers had such an impact because the studies of Mitchison and of Rajewsky had recently demonstrated a requirement for lymphocyte cooperation in the generation of secondary and primary antibody responses. Thus our Nature paper came out at a turning point in immunology. The studies of Mitchison,^20^, ^28^, ^29^ Rajewsky^21^ and Miller^25^, ^26^ led to the rapid and general acceptance of the proposition that both primary and secondary antibody responses require cooperation between B and T cells. It is important to note, though, that Claman first reported such cooperation.^27^

These circumstances set the scene for the second phase. I was now a Postdoctoral Fellow in Mel's laboratory. Mel and I talked science an average of at least an hour a day. I have never had this type of interaction with anyone before or since. We talked much more broadly than on lymphocyte activation. We were invited in 1970 to give an exposition of our views in Science, an article that went through more than 20 drafts. The article^2^ came out in September of that year. There were four main changes/extensions to the model described in our 1968 Nature paper, as a result of our mutual deliberations. We argued until we agreed upon what was most plausible. We are both responsible for what was proposed.

Firstly, we adhered to the proposition that lymphocyte cooperation was essential for lymphocyte activation and that antigen would, if at a sufficient concentration, inactivate single lymphocytes. We had no idea as to what the signals involved might be in chemical terms, so we formulated an abstract symbolism to express our thoughts. We proposed that when antigen interacts with a lymphocyte's antigen‐specific receptor a signal, signal 1, is generated that, when generated alone for a sustained time, leads to the inactivation of the lymphocyte. The activation of a responder lymphocyte, such as a B cell, also requires its antigen‐specific receptor to bind to antigen, also resulting in the generation of signal 1; its activation required the activity of helper lymphocytes that recognize the same antigen. There was no indication at this time of the MHC‐restricted recognition of antigen by T cells. The T cell receptor was envisaged to be an antibody‐like molecule. In order to make the recognition of antigen by the helper lymphocyte mandatory for the activation of the antibody precursor lymphocyte, we proposed that this recognition by the helper lymphocyte resulted in the initiation of the generation and the delivery of a second signal, signal 2, to the responder lymphocyte. We postulated that this second signal was mandatory for the activation of the responder lymphocyte, a B cell in the case of an antibody response, so that the activation of all lymphocytes was envisaged to be T helper cell‐dependent. Jacques Monod fostered this formulation of our model.

Jacques was a Non‐Resident Fellow of the Salk Institute and visited it for a few weeks in the summer of 1969. Jacques was very interested in immunology. He and Mel knew one another well. Mel had done a postdoc with Jacques, working on what became known as the lac operon at an early and critical stage of their research. I very much enjoyed Jacques' scientific company, and I think he enjoyed mine. Mel and I had ideas as to what events might constitute signal 2, as outlined in the next paragraph. Jacques suggested to us the importance, in getting our message over, that we develop a way of expressing the essence of our views, independently of the particular details of how these might be realized. We recognized the wisdom of this suggestion. It was the result of Jacque's prompting that we started to say that the interaction of antigen with the antigen‐specific receptor of the antibody precursor cell resulted in the generation of signal 1, and the recognition of a second site on the antigen, by the T helper cell, or an antigen‐specific molecule it produced, resulted in the generation of signal 2.

Our second consideration was directed at how signal 2 might be realized in cellular/molecular terms. We proposed that signal 2 was mediated by short‐range molecules, produced by the helper lymphocyte, similarly as a neuron stimulates or inhibits a cell with which it forms a synapse by the action of neural transmitters it produces, an analogy we employed at the time; alternatively, or in addition, we supposed signal 2 might involve a membrane/membrane interaction between the two interacting cells. These proposals were substantiated once interleukins were discovered and costimulatory systems established as a means by which cells of the immune system communicate. For example, it is now recognized that the activation of B cells requires both the delivery of cytokines to B cells^30^ by T helper cells and an interaction of CD40L on the surface of the activated helper T cell with the CD40 expressed by the B cell.^31^ These proposals went well beyond even indirect evidence. Nevertheless, we envisaged that only such a scenario could guarantee that the activation of a lymphocyte, for example a B cell, was helper lymphocyte‐dependent. We postulated that this proposition was essential for understanding how peripheral tolerance is achieved.

I should add a comment on the designation of the T cell required to activate a B cell as a helper T cell. Mitchison^32^ and Feldman^33^ argued that the T cells facilitated the activation of B cells, by “presenting the antigen”, but were not required, hence the designation helper. Their model was much more popular than our model for at least a decade.

Our third consideration addressed how the activity of helper T lymphocytes might be controlled. Mitchison^28^, ^29^ and later Raff^34^ showed that memory for a secondary antibody response partly resided in the carrier‐specific population of primed T cells. We postulated that precursor T helper lymphocytes could be inactivated and must be activated to provide optimal helper function for the activation of a B cell. We proposed that single helper T lymphocytes were inactivated when they interacted with antigen. In other words, the rules governing the activation and inactivation of precursor helper T lymphocytes were envisaged to be similar to those we proposed for B cell activation and inactivation. The major reason we made these proposals was because we could account, in this way, for how tolerance at the T helper cell level could be achieved.^2^ Al‐Yassin and I have recently given an account^3^ of how Weigle's experiments,^16^, ^17^ dating from the early 1960s, are most readily understood if the activation of helper lymphocytes requires lymphocyte cooperation. Weigle's studies influenced my favouring the idea in 1968/1969 that the activation of helper lymphocytes required lymphocyte cooperation.

The fourth issue was a problem, the existence of which we drew attention to. B cells are precursors of antibody‐secreting cells; their activation results in their proliferation and the differentiation of their progeny into antibody‐secreting cells. This life style of the B cell was envisaged in the formulation of the Clonal Selection Theory, as it explained not only a striking observation, namely the extreme sensitivity of in vivo immune responses to radiation of the animal around the time of immunization, but how very scarce B cells, specific for a particular antigen, could be triggered by antigen to produce substantial antibody after a lag period of a few days. Similar considerations seemed even more critical in the case of the activation of precursor helper T cells, as effector helper T cells have to be generated before B cells can be activated. Recognizing this situation, Cohn and I postulated that precursor T helper cells had to be activated by antigen to multiply and their progeny differentiate to give rise to effector helper T cells. We also proposed that lymphocyte cooperation, presumably between cooperating T cells, was required to activate T cells, thus accounting for how (peripheral) tolerance is achieved at the T cell level. In more detail, we proposed that the *optimal* antigen‐dependent activation of a responding precursor T helper cell required the activity of *effector* T helpers. This proposal led to the question of how are the first *effector T helper* cells generated? I call this problem the “priming problem”.

This type of problem is of course not unique to immunology. Life begets life, so how did life originate? Ribosomes contain proteins, and ribosomes are required for protein synthesis, so how were the first proteins produced? We shall see this “priming problem” was important in the third, last phase, of the evolution of two signal models for CD4 T cell activation. Cohn and I suggested different solutions to the priming problem.

A final point for clarity is needed. It became apparent from various observations, made after 1970, that there are at least two different processes contributing to self‐tolerance. There is central tolerance^35^ occurring in primary lymphoid organs, by self‐antigens present at sufficient levels in these organs to cause efficient clonal ablation of their corresponding lymphocytes. Other self‐antigens are more prevalent in the periphery. Lack of reactivity against such “peripheral” self‐antigens is achieved by mechanisms of “peripheral” tolerance.^5^ The two‐signal model of lymphocyte activation attempts to address how peripheral tolerance is achieved.

I would like to add that my time in Mel's laboratory as a Postdoctoral Fellow, during this second phase, was wonderful. There was a lot of lively, scientific discussion. I could think about anything I wanted to without pressure of who would provide bread the next day. As a graduate student in England I had to count my pennies. The support from my Postdoctoral Fellowships made me feel wealthy. I had the freedom to think about anything I thought interesting and important. I still think my theory of generalized autoimmunity^36^ has merit. I judge my theory of immune class regulation^6^, ^13^, ^14^, ^37^ to be as significant for basic and applied immunology as the Two Signal Model. I developed these models by myself. Mel and others in the laboratory were doing very significant work on the nature of the generation of diversity of antibody genes, both experimentally^38^ and theoretically.^39^ Alan Harris was working on the mechanism by which hydrocortisone kills lymphocytes.^40^ Other studies were directed at examining the properties of the lac repressor.^41^ We openly discussed issues in all these areas and more.

### 6.3 Introduction to the third phase

I left Cohn's laboratory in 1972. Cohn and I no longer communicated regularly. It was natural that we worked on different problems and developed different views. I naturally sought and looked out for Cohn's papers. However, I found them often difficult to understand and their contents, when I thought I understood them, often seemed implausible to me. This, on my side, naturally led me to be uncommunicative with Cohn. I believe it would have been natural, if I had liked the ideas that Cohn expressed, that we would have kept in closer contact. Until recently, and after leaving Cohn's laboratory, I only wrote a paper on the two signal model when I had something really new to say, either of a theoretical^42^ or of an experimental nature.^43^, ^44^, ^45^, ^46^, ^47^ More recently, in 2014, I decided to write a paper to reaffirm, in contemporary terms and in view of contemporary evidence, why I believed the ideas we had outlined in our 1970 Science paper were valuable and valid.^5^ This initiative on my part, as explained below, was in large measure because, though our model was supported by evidence and accepted in the context of the inactivation/activation of B cells and CD8 T cells, it was virtually ignored when it came to the inactivation/activation of CD4 T cells.

Cohn responded^7^ to my 2014 article, criticizing some central ideas I proposed. I in return responded to his comments.^48^ He felt his proposals, in the years following our 1970 Science paper, represented considerable progress,^7^ whereas I differed in this assessment.^48^ This opportunity of back and forth discussion, between Cohn and myself, is not made available by most journals. I am grateful to the Scandinavian Journal of Immunology for having been able to have this public debate.

### 6.4 The context for the third phase

Studies by Norman Klinman^49^ and by Chris Goodnow and Basten,^50^ among others, have been generally accepted as showing that antigen can inactivate B cells in the absence of helper T cells. Studies by Keene and Forman,^51^ among others, show that the activation of some CD8 T cells, as assessed by their priming, requires CD4 T helper cells. The CD4 T cell and CD8 T cell had to recognize antigen presented by the same APC. In this sense, the interaction required “the recognition of linked epitopes”. Guerder and Matzinger, among others, showed that antigen could inactivate such CD8 T cells in the absence of CD4 T cells.^52^ All these observations were consistent with the postulates of our 1970, Two Signal Model.^2^

Studies by Lafferty^53^ and others led to the proposal that the activation of a CD4 T cell required an antigen‐mediated signal, signal 1, and a second, costimulatory signal, or signal 2, delivered by the APC, the archetypal such signal occurring when the T cell's CD28 molecules interact with B7 molecules on the APC. This model was a critical step in the evolution of ideas on CD4 T cell activation. Lafferty credited Bretscher and Cohn with the idea that T cell activation requires two signals,^54^ but Lafferty's envisaged costimulatory (CoS) signal was critically different from “our” signal 2. Our proposal was that signal 2 is generated when a helper lymphocyte recognizes antigen and consequently delivers a signal to a responding, precursor helper T cell. Thus our 1970 proposal was that helper lymphocyte activation required T cell cooperation.

Charlie Janeway recognized that the presence of helper T cells was required to activate B and CD8 T lymphocytes and that antigen could inactivate these lymphocytes in the absence of helper T cells. He therefore, in 1989, naturally focussed his attention on the circumstances that lead antigen to either inactivate or activate CD4 T helper cells. As is well known, he postulated that the expression of the critical CoS molecule on the APC, needed to generate the second or CoS signal and required to activate CD4 T cells, was only upregulated if a pattern recognition receptor (PRR) of the APC recognizes a pathogen‐associated molecular pattern (PAMP). In the absence of an appropriate PAMP, and consequently inadequate expression of the critical CoS molecules, antigen is envisaged to inactivate the CD4 T cell.^55^ This was a radical proposal. Janeway initially suggested that an appropriate description of his novel proposal was that the immune system distinguishes between non‐infectious self from infectious non‐self, rather than self from non‐self.^56^ I do not think this was an appropriate description of his proposal. His proposal is better described as discrimination between infectious and non‐infectious entities. Janeway never addressed how non‐self, non‐infectious antigens, such as foreign vertebrate antigens, not expressing PAMPs, could be immunogenic. I considered and consider his proposal to be a radical and an unwarranted change in perspective. I understand from Cohn's papers he shared my view.^7^ Matzinger pointed out that allogenic grafts, anticipated not to express PAMPS, were rejected. This and other considerations led her to propose that the induced expression of the CoS molecules, by the APC and required to activate CD4 T cells, only occurred under diverse, stressful conditions, collectively described as “danger”.^57^ The most popular, contemporary view appears to be an amalgam of Janeway's and Matzinger's ideas, according to which either PAMP‐dependent or danger‐associated molecular pattern (DAMP)‐dependent signals are required to upregulate the critical costimulatory molecules needed for CD4 T cell activation.

Three major and related advances post‐1970 had an impact on our ideas, without undermining them. Firstly, the roles of processing and presentation of antigen by antigen presenting cells (APC) were clarified. Secondly, the fact that nominal antigen is processed and its peptides presented on MHC molecules fitted in with the realization that the recognition of antigen by the T cell receptor is MHC‐restricted.^58^ Thirdly, the inadequacy of the antigen‐bridge model for how B cells interact with helper T cells became apparent.

Lanzavecchia's observations, together with these advances, led him to propose a new model of the B cell/helper T cell interaction. He employed a hapten (h) carrier system and studied the requirements to induce anti‐hapten antibody. He postulated that an anti‐h B cell endocytoses an h‐Q conjugate, where Q represents the carrier protein; the conjugate is then processed inside the B cell, and the peptide q/MHC complexes are subsequently presented by the B cell. The T helper cell specific for the nominal antigen Q can then recognize these peptide q/MHC complexes and deliver signal 2. The critical point is that the anti‐h B cell would not present peptide q/MHC complexes in the presence of Q and hR, but rather it would present peptide r/MHC complexes. The hapten must be coupled to Q if the anti‐h B cell is to endocytose Q via its antigen‐specific receptors.^59^ Thus, Lanzavecchia's model explained why successful B cell/helper T cell interaction requires the “linked recognition of antigen”.

### 6.5 The problem of linked recognition in the cooperation between CD4 T cells

Somewhat similarly, our extensive studies on the requirements to activate CD4 T cells show that the activation of Q‐specific responder T helper cells can be facilitated by R‐specific T helper cells in the presence of the conjugate Q‐R, but not when Q and R are present as separate molecules.^43^, ^44^, ^45^, ^46^, ^47^ This requirement for linkage is physiologically critical. It ensures that the activation of a responder CD4 T cell specific for a peripheral self‐antigen, pS, cannot be facilitated by a helper T cell specific for a foreign antigen, F that does not crossreact with pS, in the presence of F. It seemed, if linkage between R and Q was not required to facilitate the activation of a responder, Q‐specific helper T cell, that responses to peripheral self‐antigens would be facilitated by helper T cells specific for irrelevant, foreign antigens.^3^, ^13^ As already indicated, the extensive studies by my students and myself strongly support the idea that the activation of responding helper T cells can be optimally facilitated by effector helper T cells acting via the recognition of linked epitopes. These experiments,^43^, ^44^, ^45^, ^46^, ^47^ and others by other researchers,^60^, ^61^ were consistent with our 1970 ideas on the requirements to activate CD4 T cells. They were not anticipated by Janeway's or Matzinger's models. I proposed in 1999 that the requirement for linkage could be ensured if the APC‐mediating CD4 T cell cooperation is an antigen‐specific B cell.^42^ Thus, the cooperation between Q‐specific CD4 T cells and R‐specific CD4 T cells, mediated by a Q‐R conjugate, is envisaged to involve a Q‐ or R‐specific B cell acting as an APC and is supported by observation.^47^, ^62^ I envisaged a role for other APC, such as DC or macrophages, in a first step, in which antigen stimulates CD4 T cells to divide, before the B cell‐mediated CD4 T cell cooperation, constituting a second step, takes place.^13^, ^14^, ^42^ I postulated that CD4 T cells only completing step one are, in time, inactivated.

Cohn disagreed with this solution for how linked recognition between cooperating CD4 T cells might be realized, and has discussed other possibilities,^7^ that I think implausible.^48^ I do not discuss these issues further as they have been the subject of recent debate.

### 6.6 The priming problem

We had proposed that effector helper T cells are required for antigen to optimally activate responder T helper cells, thus generating more effector helper T cells. How then are the first effector helper T cells generated? We refer to this as the priming problem.

I proposed in a 1972 invited article,^63^ while still in Cohn's laboratory, a solution to this problem. I envisaged that precursor helper T cells might have a low but significant level of effector helper activity. In this case, the activation of precursor helper T cells could be initiated by substantial antigen‐dependent cooperation between precursor CD4 T helper cells. This cooperation was envisaged to give rise to T helper cells with full effector activity. In this case, the understanding we thought we had, of how the one lymphocyte/multiple lymphocyte model for the antigen‐dependent inactivation/activation of lymphocytes, could explain peripheral tolerance, still held. Thus, this proposal was conservative of our primary ideas expressed in our 1970 Science article. I still think this to be the most plausible solution, a solution we are currently assessing.

Cohn stated^7^ that the priming problem was not addressed until he made a proposal in 1983,^64^, ^65^ despite my proposal of 1972.^63^ He proposed that precursor helper T cells are activated and inactivated as outlined in our 1970 Science paper but, in addition, proposed a new pathway to generate the first effector helper T cells during development. He proposed that precursor T helper cells could, *in the absence of antigen*, *slowly* differentiate into effector helper T cells. Cohn envisioned that this pathway would not happen for CD4 T cells specific for self‐antigens, as self‐antigens are always present and their corresponding precursor helper T cells would be *quickly* inactivated by the mechanisms incorporated in our two‐signal model.^64^ In contrast, a foreign antigen, F, is different from self‐antigens in not always being present. When not present during development or in very young animals, effector CD4 T cells specific for F would be generated by this antigen‐independent mechanism. Cohn states, “it is the sufficiency or insufficiency of effector T‐helpers (eTh) that determines responsiveness”.^7^ According to this proposal, the generation of the first effector helper T cells is both antigen and lymphocyte cooperation independent, and the presence of these effector T cells in a mature individual is the critical factor in initiating an immune response. Thus, this proposal^64^, ^65^ is radically different from what we proposed in our 1970 Science paper, according to which a quorum of lymphocytes is needed to initiate the generation of a response. The proposal may be attractive, but should not be confused with our 1970 proposal and should be judged on its own merits. I have recently discussed why I feel this proposal to be implausible.^48^ Most importantly, it appears that a quorum of lymphocytes is required to activate T cells,^3^ consistent with our 1970 model, but not Cohn's proposal for how the priming problem may be resolved.

## 7 RESPONSE TO REVIEWERS' COMMENTS

I would like to thank the four reviewers for their careful and considered comments. There is no doubt that these comments allowed me to improve the manuscript. Two comments made by different reviewers call for a response.

One comment by one reviewer was: “What about B cell deficiency, where the (human) patient altogether lacks B cells? If B cells are important for providing the second antigen specific signal, how can these patients have a relatively adequate defense against viruses? Some practical examples based on current immunological thinking …would have made this account much more relevant and interesting.” My initial response to this comment, before discussing the science, is to say that the primary purpose of the article was to give my perspective on the history of a model that has remained pertinent for almost 50 years.

Now for the science, I personally never expect all observations to fit into one picture, as no picture is complete, and because some of the frameworks we employ in “obtaining” our observations are likely wrong. The evidence for a mandatory role of B cells in generating murine immune responses appears to be highly contradictory. What to do about this situation was indeed the subject of recent discussions between Cohn and myself.^7^, ^48^ I briefly illustrate my view. It is reported both that B cell KO mice can generate immune responses,^66^ and that patients with cell‐mediated autoimmunity can be treated successfully by depleting their B cells.^67^, ^68^, ^69^ These findings respectively contradict and support the view that B cells have an essential role as APC in cell‐mediated responses. How to face this dilemma? I would suggest B cell KO mice may not reflect normal physiology, given that the absence of B cells may affect these animals in multiple ways.^48^ This consideration, I suggest, is pertinent to the reviewer’ comments.

Another reviewer felt the omission of discussing the role of co‐inhibitory signals, in the negative control of lymphocyte activation, was unbalanced. My perspective is that whether the one lymphocyte/multiple lymphocyte model holds is a fundamental question. This minimal model can accommodate a requirement or a lack of requirement for a co‐inhibitory signal in lymphocyte inactivation. What is particularly appealing to me is the clear role of co‐inhibitory signals in feedback regulation. I believe Sinclair originally proposed that antigen/IgG complexes could mediate an Fc‐mediated, negative signal to B cells, requiring the antigen to also bind to the B cell's antigen‐specific receptors.^70^ This constitutes an elegant form of antigen‐specific feedback regulation of antibody production. This negative signal is the archetypal co‐inhibitory signal.^71^ A mechanism of T cell activation requiring T cell collaboration, as Cohn and I proposed, would obviously be an explosive situation in the absence of feedback, as exemplified by the phenotype of CTLA4 KO mice.^72^ I am not convinced a co‐inhibitory signal is required to inactivate naïve T cells. The physiological significance of co‐inhibitory signals, as a feedback mechanism, is evident, but their potential physiological role in inactivation of T cells is less evident to me, or their supposed existence called for by observation.

One reviewer pointed out that Cohn had a paper coming out in this journal, entitled ‘The real “danger” lies in failure to confront fundamentals',^73^ and suggested I might comment on it. This paper primarily addresses the issue of the mechanism of self‐nonself discrimination, with a central role for the one lymphocyte/multiple lymphocyte model for the antigen‐dependent inactivation/activation of lymphocytes. I find it inappropriate and sad that Cohn did not cite our 1968 Nature^11^ or 1970 Science papers,^2^ where this and the two signal model were first proposed.

## 8 CONCLUDING COMMENT

Mel Cohn died in October 2018. A first version of this article was submitted before Cohn's death. His death occurred during the reviewing process. I asked the Associate Editor, who was dealing with this manuscript, if he could delay its consideration for a few months.

I wish to give a brief explanation of the way I have responded to what I perceive as inaccuracies in the history Cohn gave of his and my involvement in the formulation of The Two Signal Model of Lymphocyte Activation. I have described above why Cohn's 1994 Wisdom of Hindsight article^4^ disturbed me, when it first appeared, concerning this history. I had also felt that the coherence of our 1970 theory was undermined by Cohn's subsequent articles, in which he felt^7^ he was improving the theory. Cohn stated, in his 1994 paper on his contributions to immunology: “The ‘two signal model’ had a rocky intellectual history; but, as formulated today, it is highly likely to be correct. In essence, there is at present no validly competing model”.

For years, I did not criticize in print Cohn's revisions or his further development of our ideas. I thought it more worthwhile that my students and I concentrate on experimentally testing predictions of the model we favoured,^2^, ^42^ and to support our view in the discussion sections of our experimental papers. However, our considerable experimental studies,^43^, ^44^, ^45^, ^46^, ^47^ on the mechanism by which CD4 T cells cooperate in the activation of CD4 T cells, were pretty well ignored by the immunological community, with the ascendancy of Janeway's PAMP and Matzinger's Danger Models.

My 2014 article,^5^ reaffirming the plausibility of our 1970 ideas in a contemporary context, drew an extended response from Cohn.^7^ This response was critical of some of my proposals and made some statements on the history of our Two Signal Model that I felt I should challenge. I decided to respond to the scientific criticisms in another “Discussion Forum” article.^48^ I also decided, at this stage, 44 years after our Science paper, to write my personal recollection of the history of how the Two Signal Model came to be formulated, and to post it on my website. However, before doing so, I emailed, in May 2015, a draft of my perspective of this history to Cohn, asking him to give me any comments he felt to be appropriate. He did not initially respond to this email, so I posted my perspective. Shortly after this posting, Cohn emailed me. He did not express any disagreement over or endorse the facts I recalled. I wish readers to know that I did attempt to initiate a dialogue with Cohn, concerning our differences over the history of the Two Signal Model, when this was still possible.
